# Supplementary material for: Syntheses of l-Rhamnose-Linked Amino Glycerolipids and Their Cytotoxic Activities against Human Cancer Cells
Source: Molecules. 2020 Jan 28;25(3):566. doi: 10.3390/molecules25030566 (PMC7037447; doi:10.3390/molecules25030566)
Supplement: Supplementary file 1 [file molecules-25-00566-s001.pdf]

molecules

MDPI

Supplementary Materials

Syntheses of L-Rhamnose-Linked Amino Glycerolipids and Their Cytotoxic Activities against Human Cancer Cells

Makanjuola Ogunsina [1], Pranati Samadder [2], Temilolu Idowu [1], Mark Nachtigal [2,3,4], Frank Schweizer [1,*] and Gilbert Arthur [2,*]

[1] Department of Chemistry and Biochemistry, Faculty of Science, University of Manitoba, Winnipeg, MB, R3T 2N2, Canada; mogunsina@gmail.com (M.O.); idowut@myumanitoba.ca (T.I.)
[2] Department of Biochemistry and Medical Genetics, Rady Faculty of Health Sciences, University of Manitoba, Winnipeg, MB, R3E 0W9, Canada; sohagdalkutir@gmail.com (P.S.); Mark.Nachtigal@umanitoba.ca (M.N.)
[3] Department of Obstetrics, Gynecology and Reproductive Sciences, Rady Faculty of Health Sciences, University of Manitoba, Winnipeg, MB, R3E 0W9, Canada
[4] Research Institute in Oncology & Hematology, CancerCare Manitoba, Winnipeg, MB, R3E 0V9, Canada
* Correspondence: Frank.Schweizer@umanitoba.ca (F.S.); Gilbert.Arthur@umanitoba.ca (G.A.)

Received: 4 December 2019; Accepted: 26 January 2020; Published: date

Abstract: A major impediment to successful cancer treatment is the inability of clinically available drugs to kill drug-resistant cancer cells. We recently identified metabolically stable L-glucosamine-based glycosylated antitumor ether lipids (GAELs) that were cytotoxic to chemotherapy-resistant cancer cells. In the absence of commercially available L-glucosamine, many steps were needed to synthesize the compound and the overall yield was poor. To overcome this limitation, a facile synthetic procedure using commercially available L-sugars including L-rhamnose and L-glucose were developed and the L-GAELs tested for anticancer activity. The most potent analog synthesized, 3-amino-1-*O*-hexadecyloxy-2R-(*O*–α-L-rhamnopyranosyl)-*sn*- glycerol **3**, demonstrated a potent antitumor effect against human cancer cell lines derived from breast, prostate, and pancreas. The activity observed was superior to that observed with clinical anticancer agents including cisplatin and chlorambucil. Moreover, like other GAELs, **3** induced cell death by a non-membranolytic caspase-independent pathway.

Keywords: glycosylated antitumor ether lipids; L-rhamnose-based glycolipids; chemotherapy resistant; caspase independent

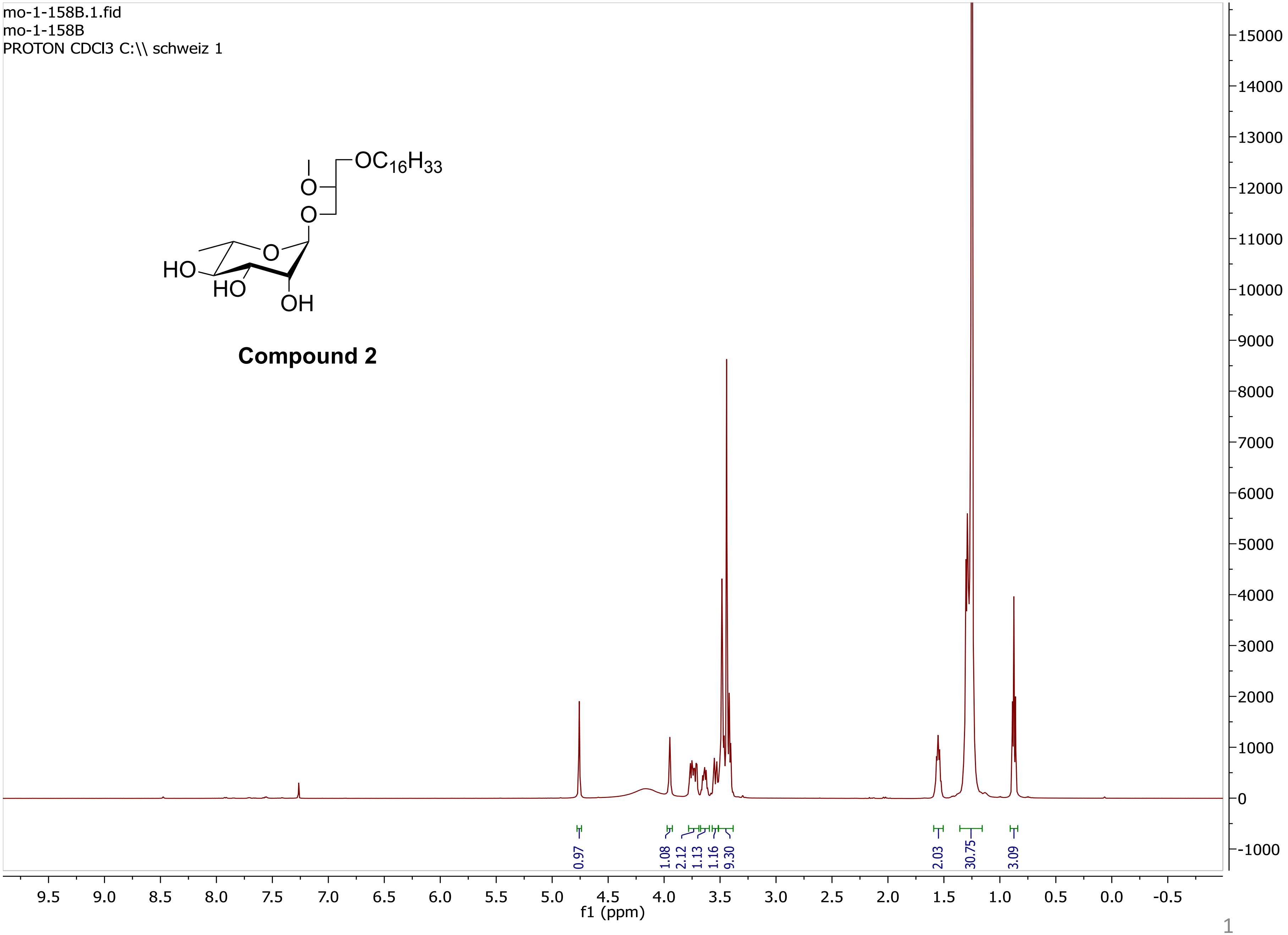
mo-1-158B.1.fid
mo-1-158B
PROTON CDCl3 C:\\ schweiz 1
OC16H33
O
O
O
HO
HO
OH
Compound 2
0.97
1.08
2.12
1.13
1.16
9.30
2.03
30.75
3.09
f1 (ppm)

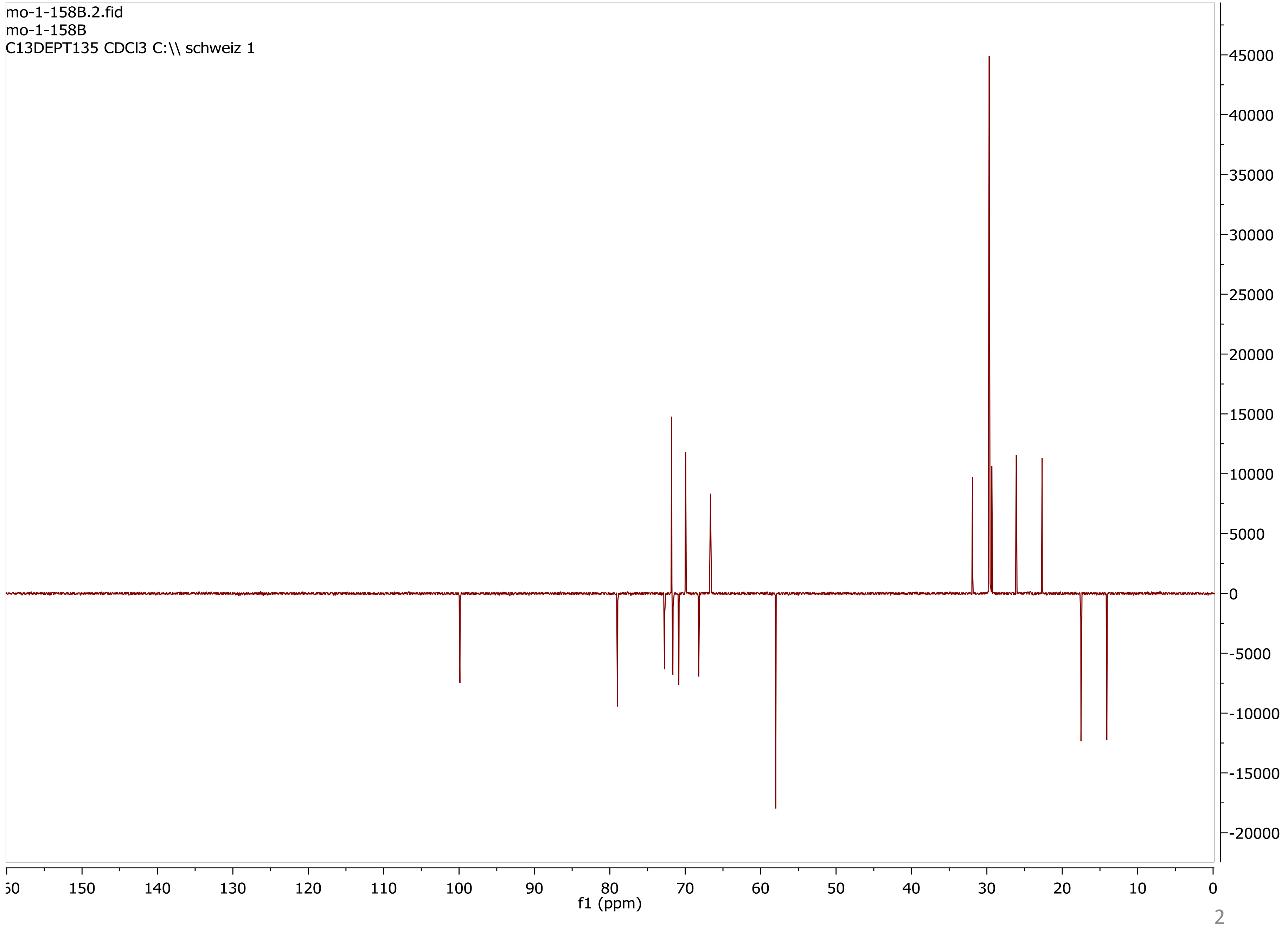
mo-1-158B.2.fid
mo-1-158B
C13DEPT135 CDCl3 C:\\ schweiz 1
f1 (ppm)

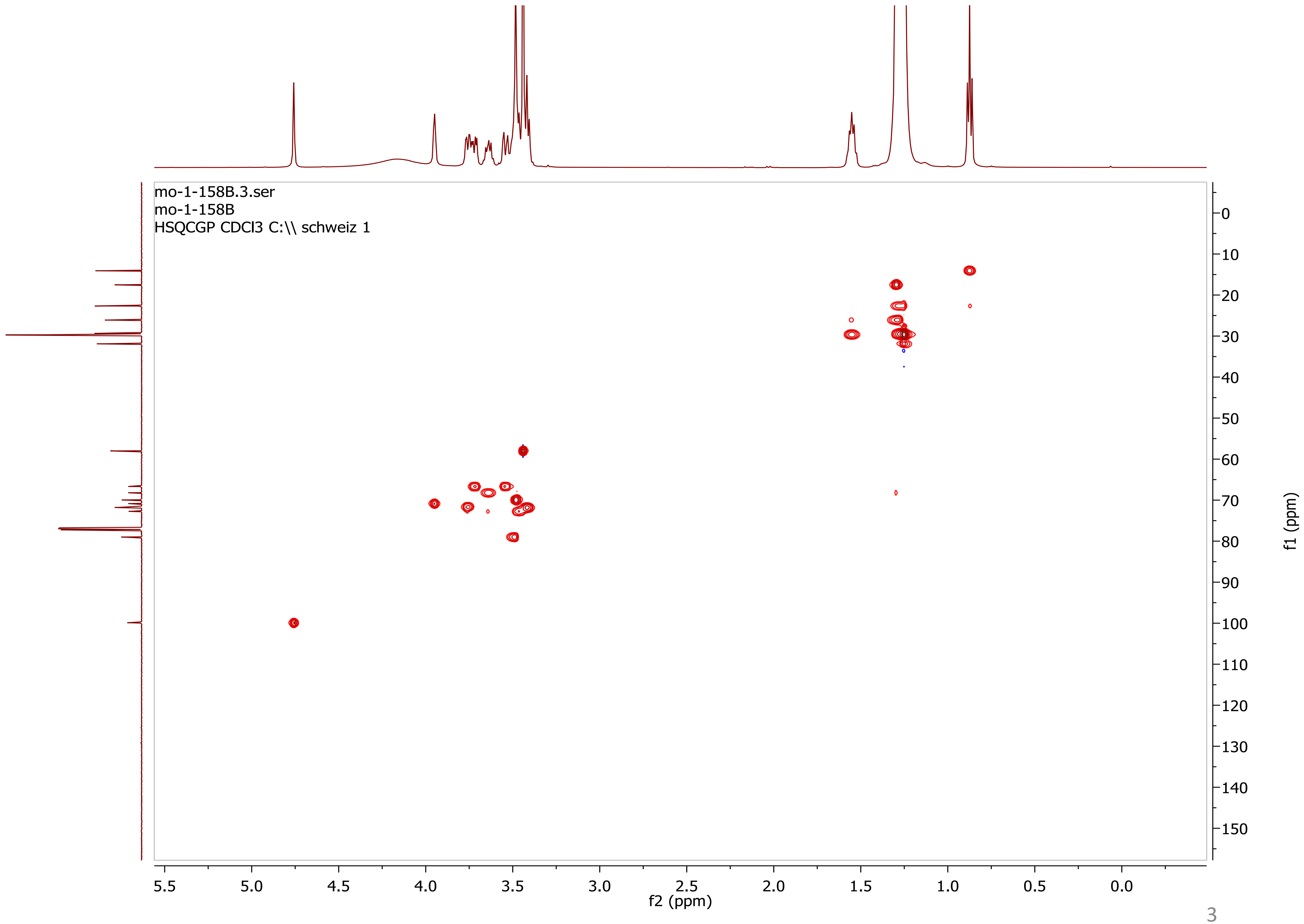
mo-1-158B.3.ser
mo-1-158B
HSQCGP CDCl3 C:\\ schweiz 1
f2 (ppm)
f1 (ppm)
5.5
5.0
4.5
4.0
3.5
3.0
2.5
2.0
1.5
1.0
0.5
0.0
0
10
20
30
40
50
60
70
80
90
100
110
120
130
140
150

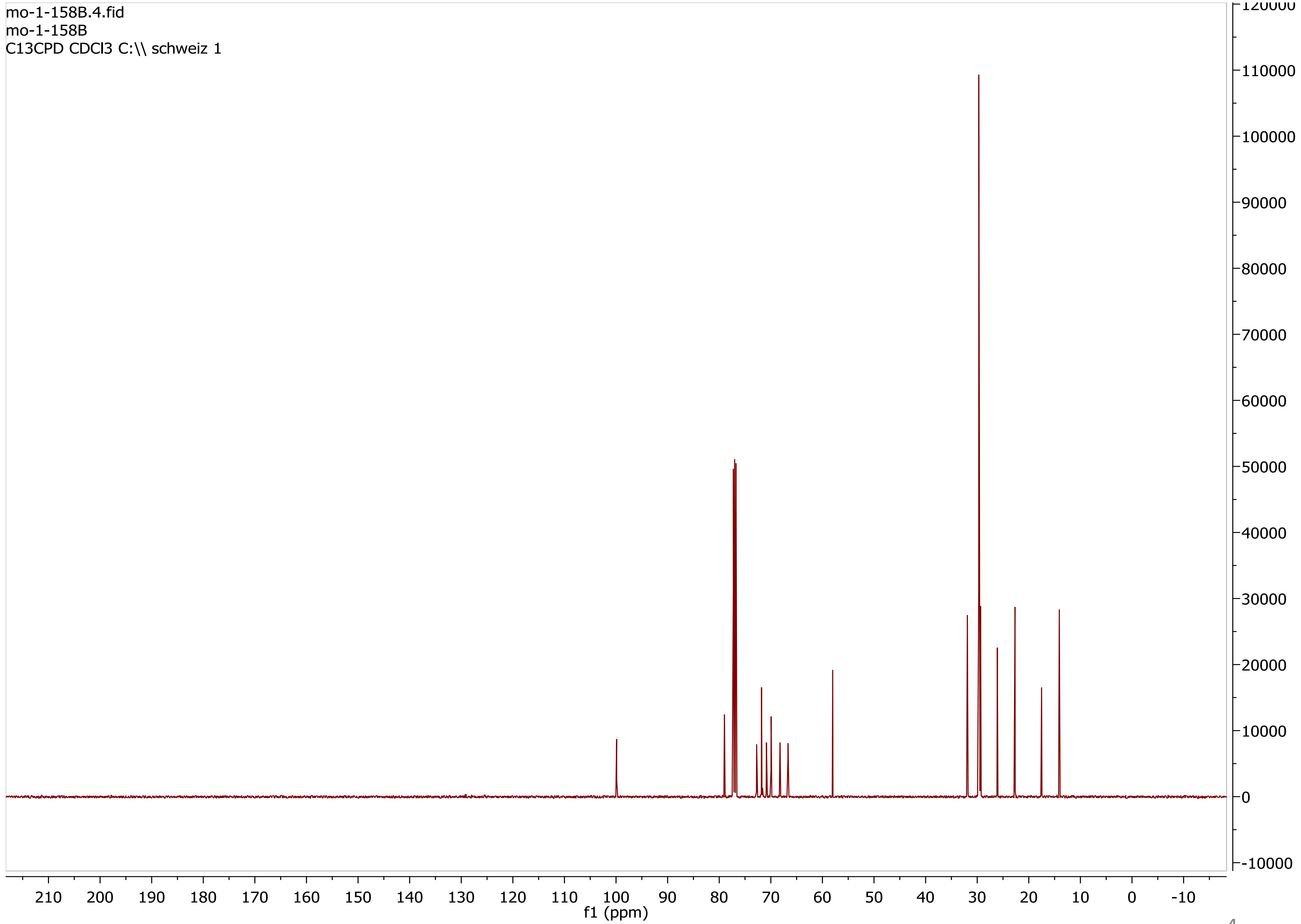
mo-1-158B.4.fid
mo-1-158B
C13CPD CDCl3 C:\\ schweiz 1
f1 (ppm)

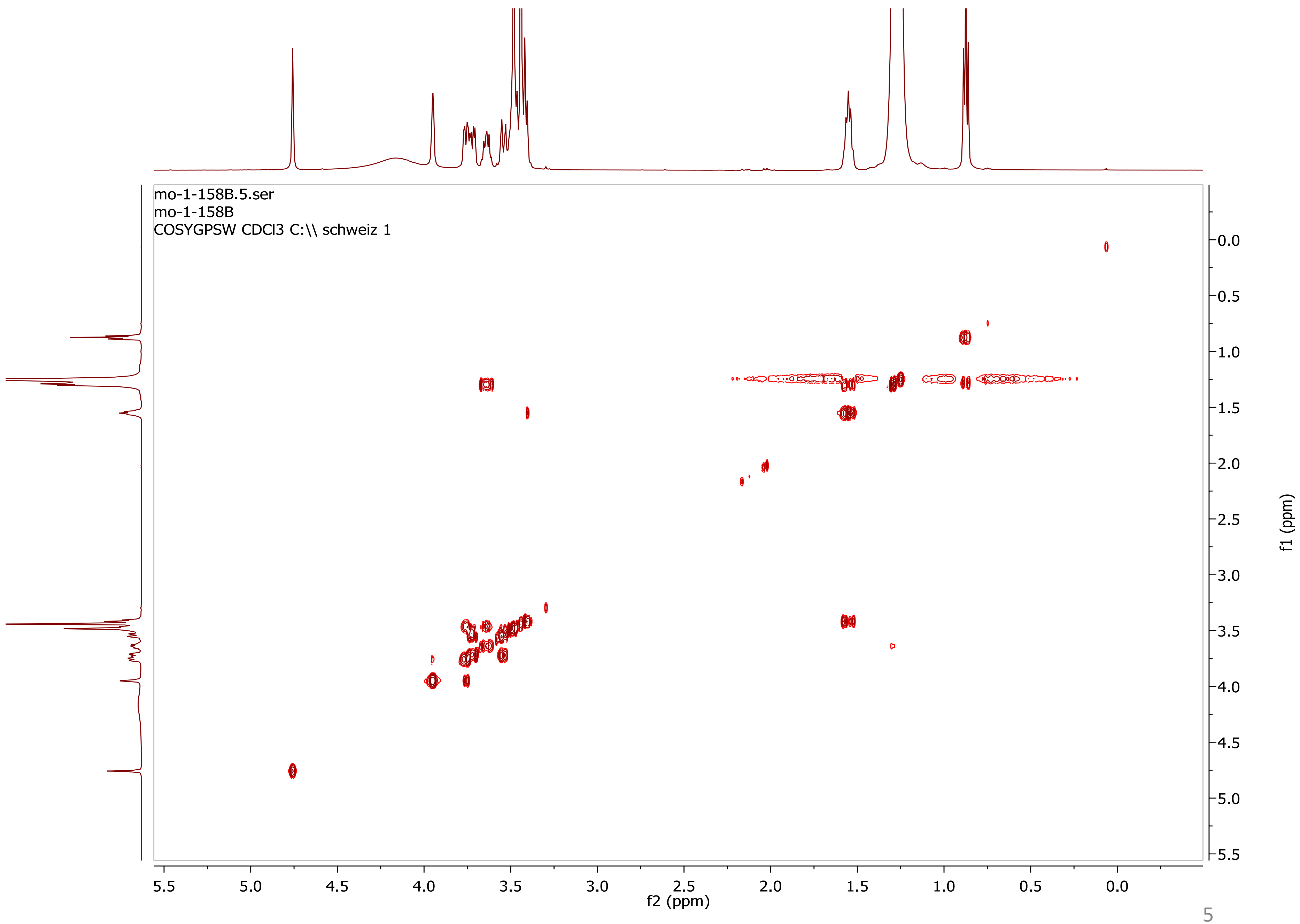

mo-1-158B.5.ser
mo-1-158B
COSYGPSW CDCl3 C:\\ schweiz 1
f2 (ppm)
f1 (ppm)

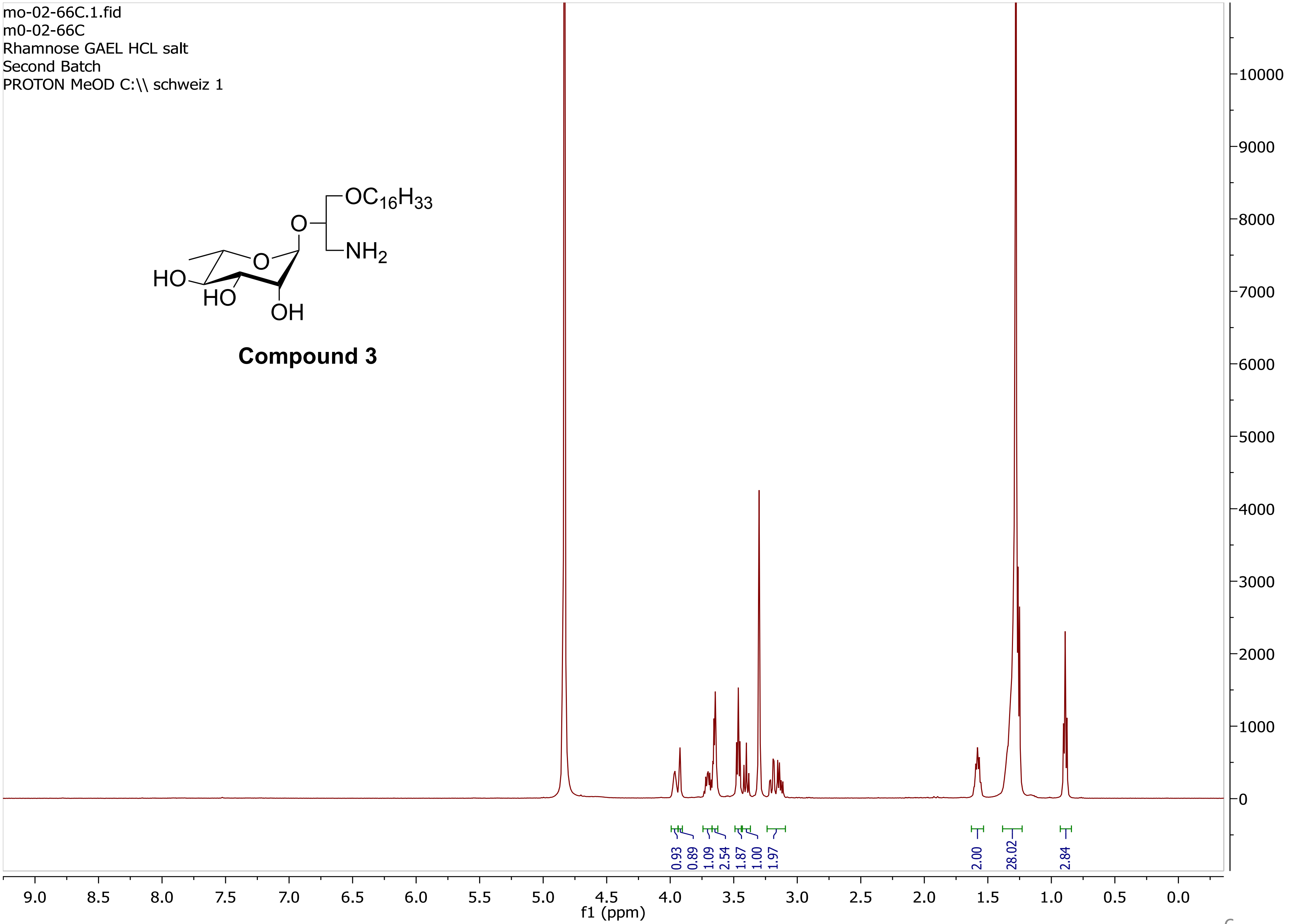
mo-02-66C.1.fid
m0-02-66C
Rhamnose GAEL HCL salt
Second Batch
PROTON MeOD C:\\ schweiz 1
$OC_{16}H_{33}$
O
NH_2
O
HO
HO
OH
Compound 3
0.93
0.89
1.09
2.54
1.87
1.00
1.97
2.00
28.02
2.84
f1 (ppm)

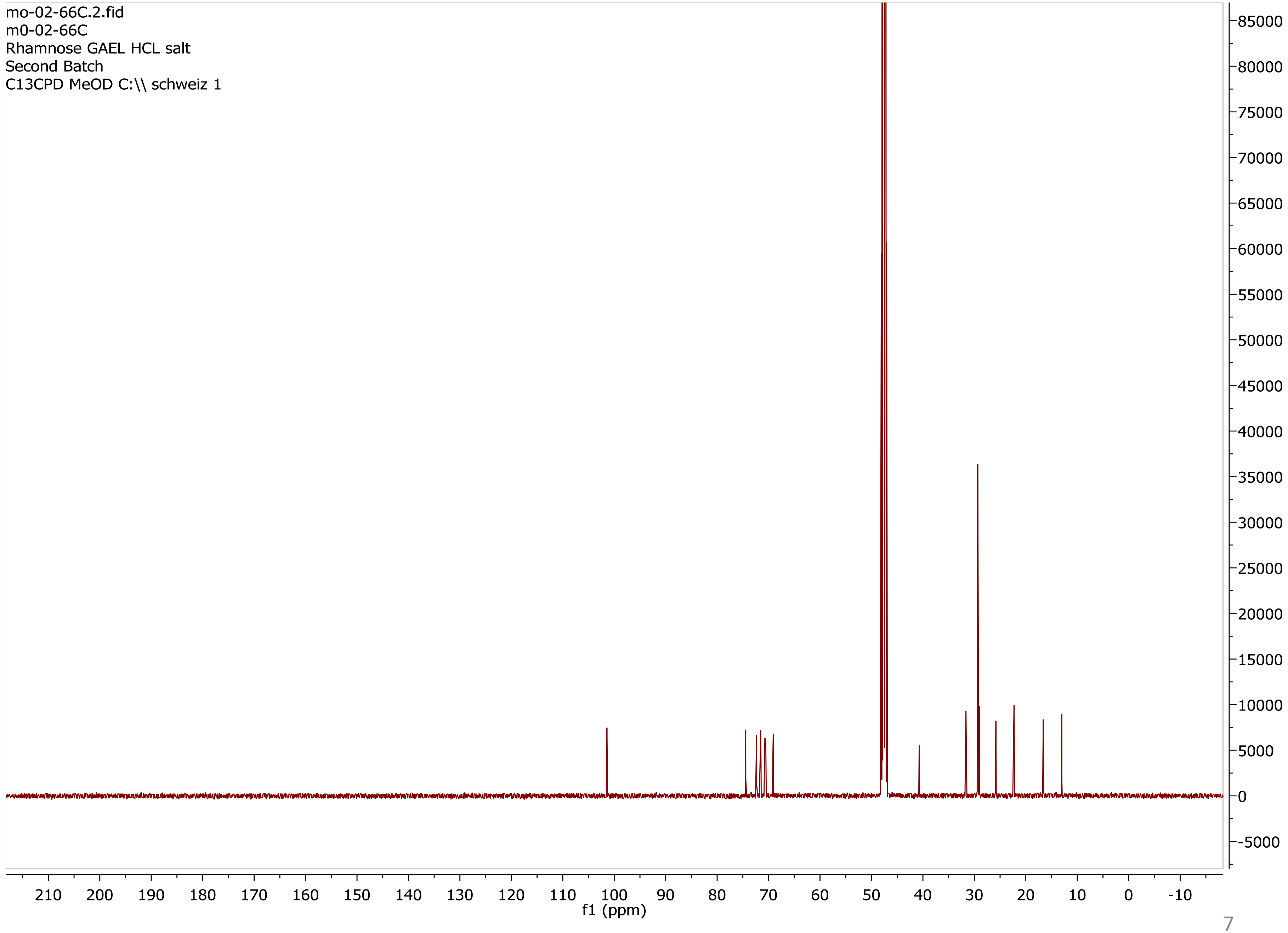

mo-02-66C.2.fid
m0-02-66C
Rhamnose GAEL HCL salt
Second Batch
C13CPD MeOD C:\\ schweiz 1
f1 (ppm)

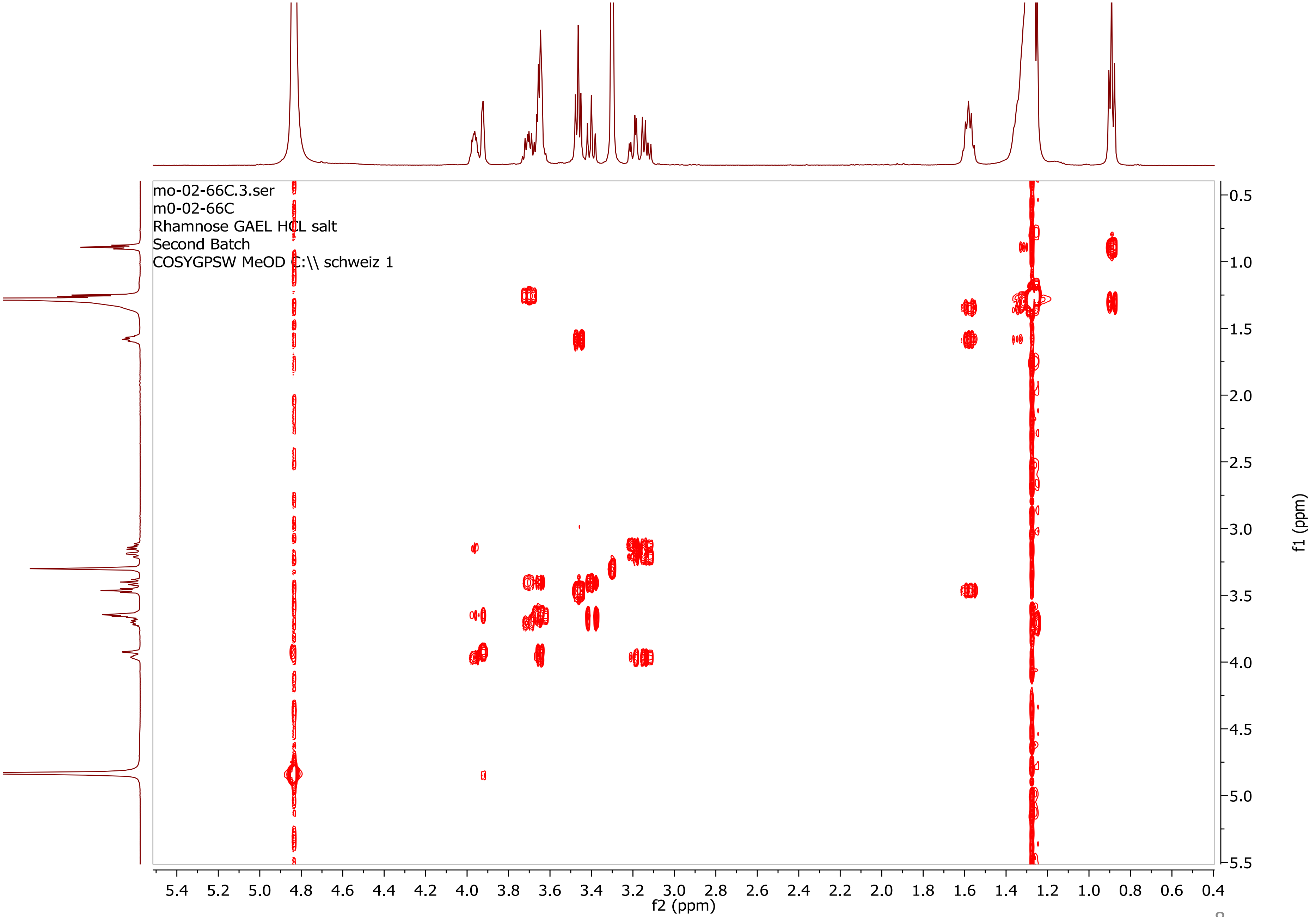

mo-02-66C.3.ser
m0-02-66C
Rhamnose GAEL HCL salt
Second Batch
COSYGPSW MeOD C:\\ schweiz 1
f2 (ppm)
f1 (ppm)
5.4 5.2 5.0 4.8 4.6 4.4 4.2 4.0 3.8 3.6 3.4 3.2 3.0 2.8 2.6 2.4 2.2 2.0 1.8 1.6 1.4 1.2 1.0 0.8 0.6 0.4
0.5 1.0 1.5 2.0 2.5 3.0 3.5 4.0 4.5 5.0 5.5

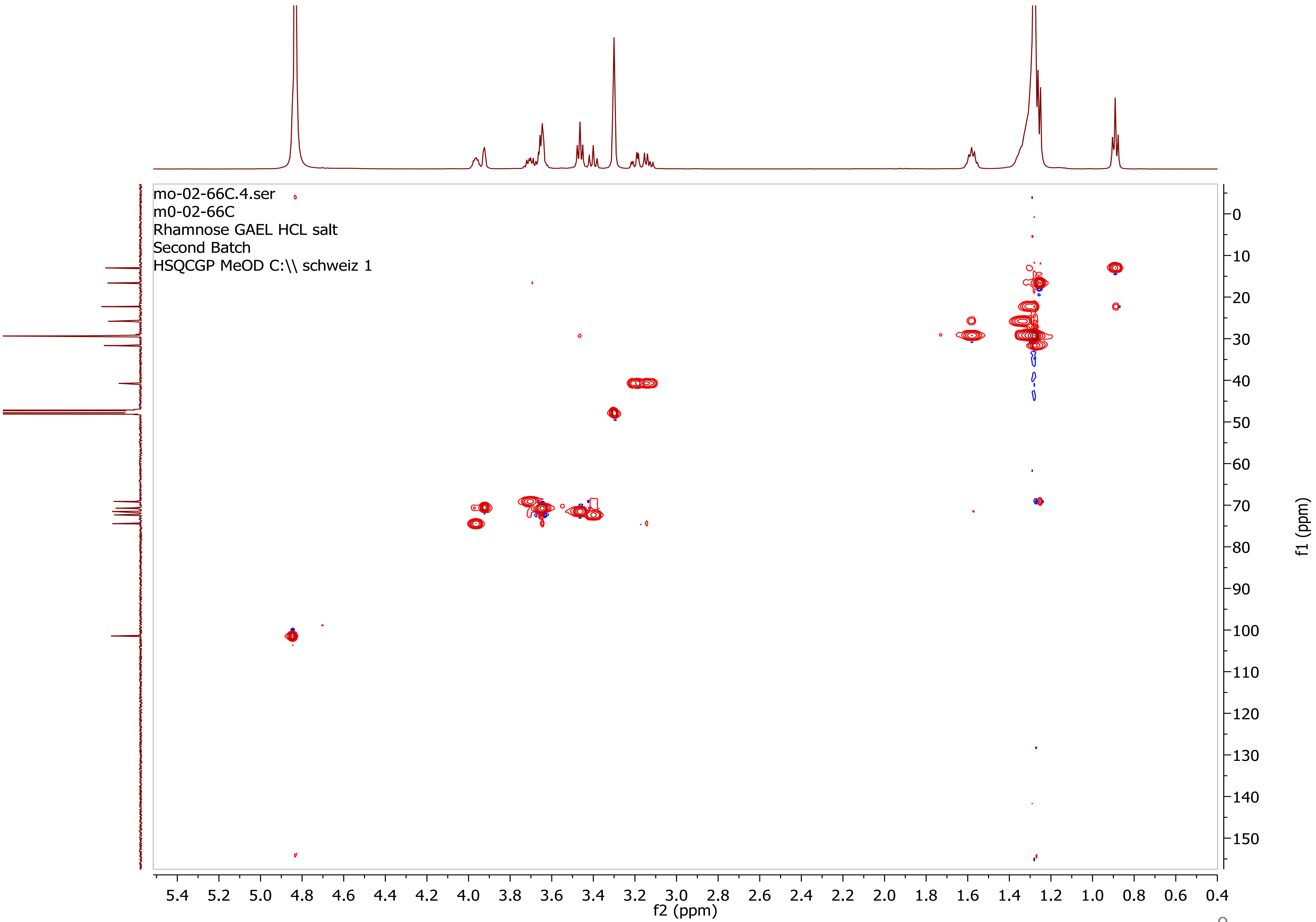
mo-02-66C.4.ser
m0-02-66C
Rhamnose GAEL HCL salt
Second Batch
HSQCGP MeOD C:\\ schweiz 1
f2 (ppm)
f1 (ppm)

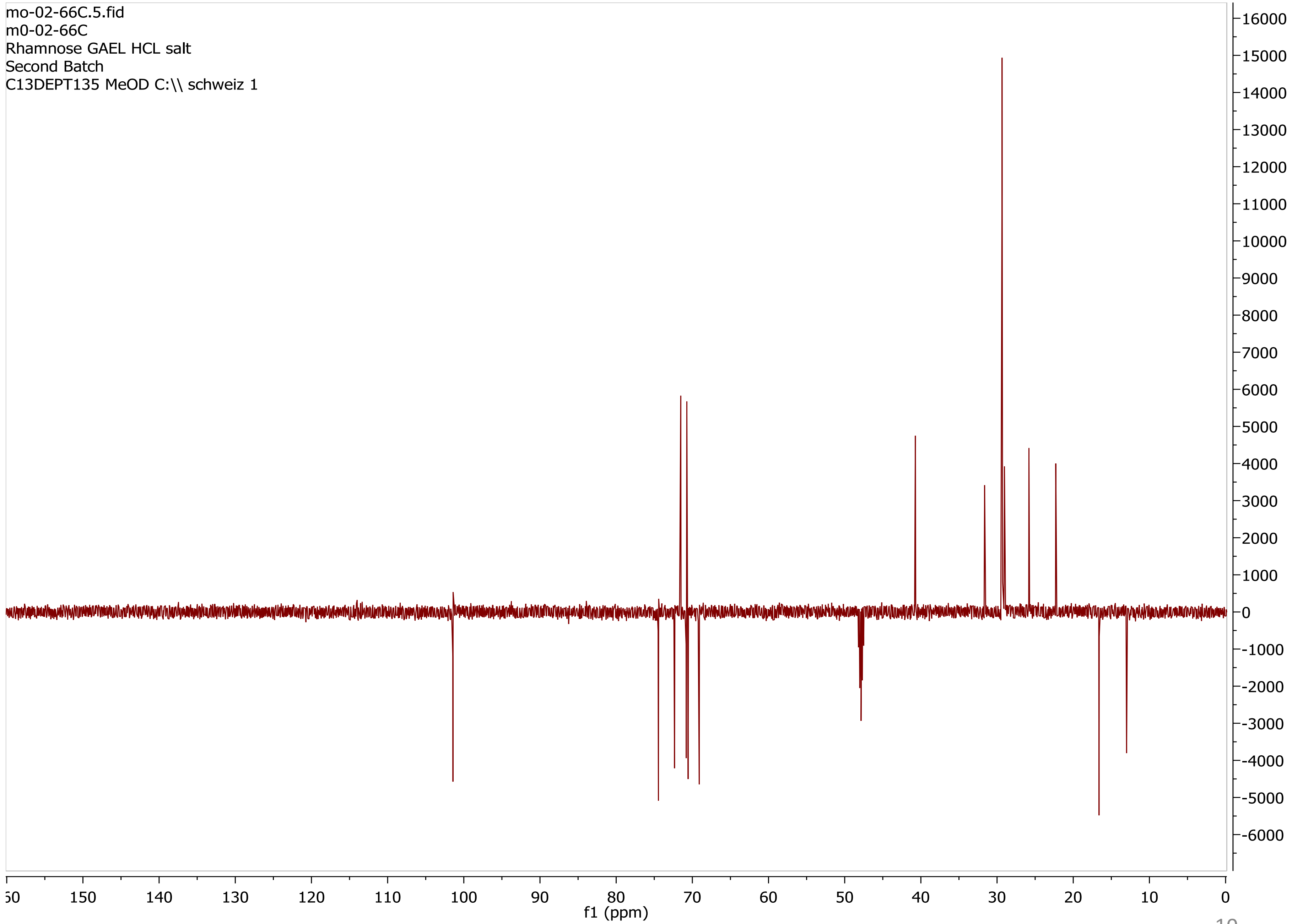
mo-02-66C.5.fid
m0-02-66C
Rhamnose GAEL HCL salt
Second Batch
C13DEPT135 MeOD C:\\ schweiz 1
f1 (ppm)

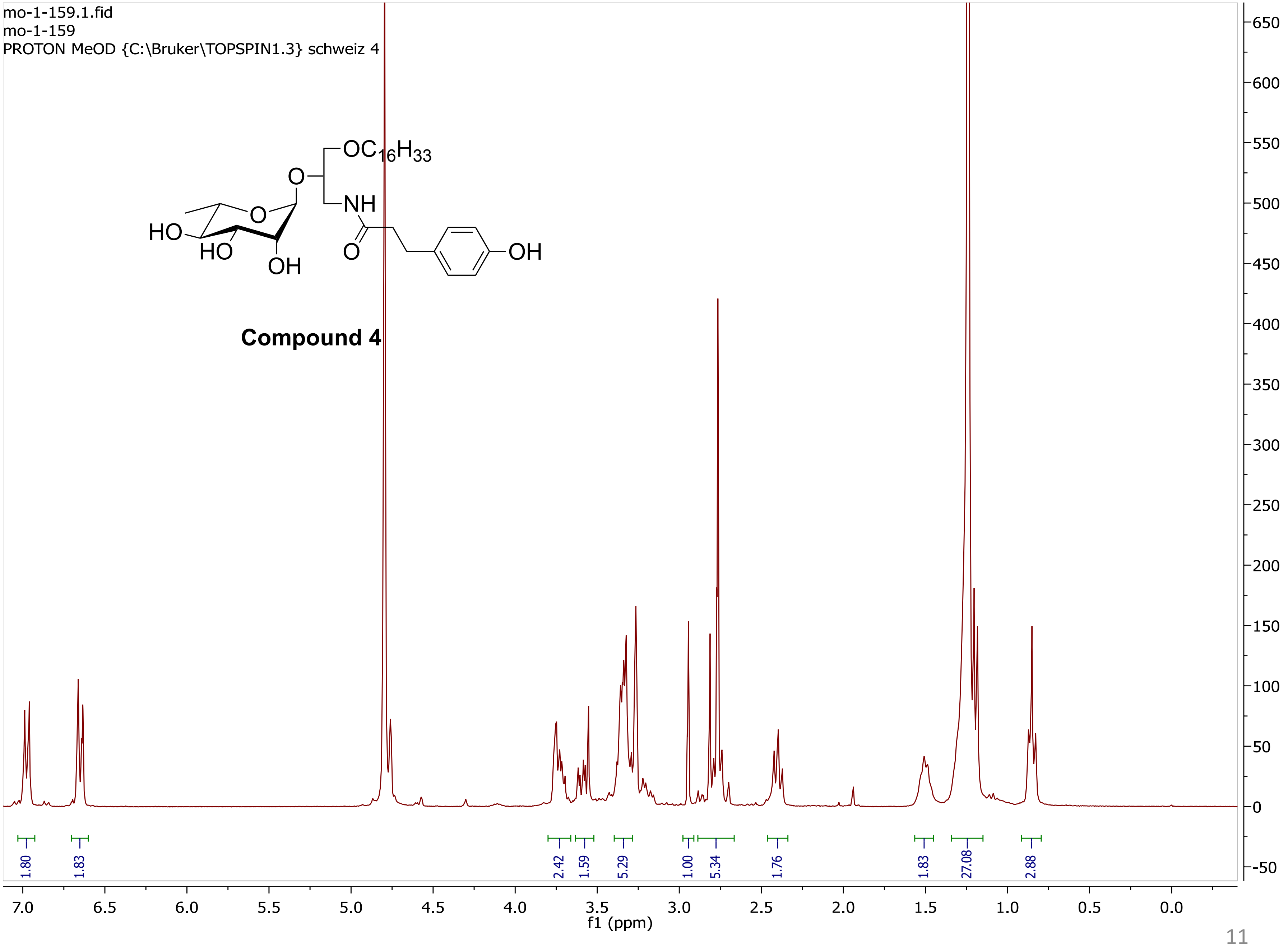
mo-1-159.1.fid
mo-1-159
PROTON MeOD {C:\Bruker\TOPSPIN1.3} schweiz 4
OC16H33
O
NH
HO
HO
OH
O
OH
Compound 4
1.80
1.83
2.42
1.59
5.29
1.00
5.34
1.76
1.83
27.08
2.88
7.0
6.5
6.0
5.5
5.0
4.5
4.0
3.5
3.0
2.5
2.0
1.5
1.0
0.5
0.0
f1 (ppm)
650
600
550
500
450
400
350
300
250
200
150
100
50
0
-50

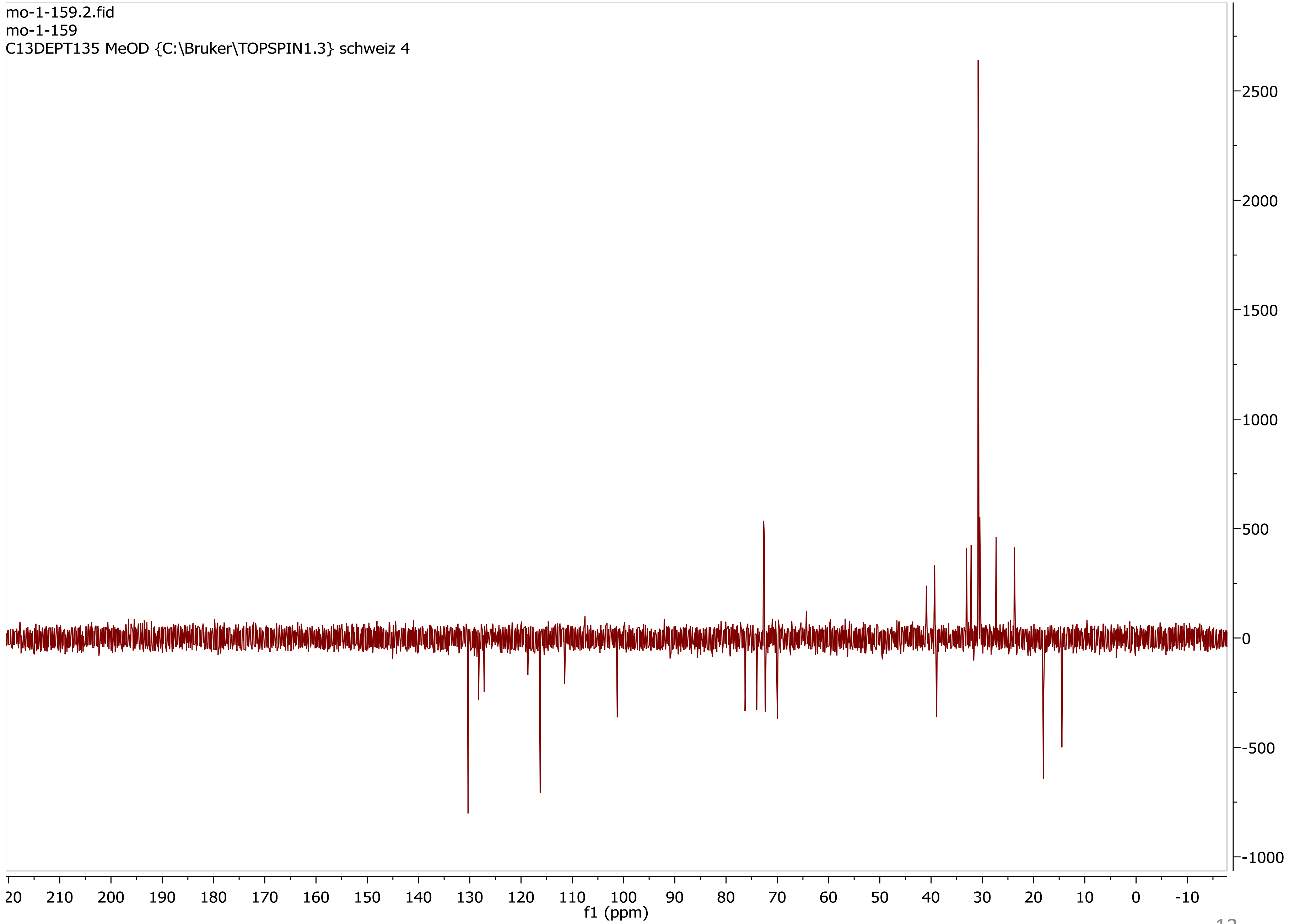

mo-1-159.2.fid
mo-1-159
C13DEPT135 MeOD {C:\Bruker\TOPSPIN1.3} schweiz 4
2500
2000
1500
1000
500
0
-500
-1000
20
210
200
190
180
170
160
150
140
130
120
110
100
90
80
70
60
50
40
30
20
10
0
-10
f1 (ppm)

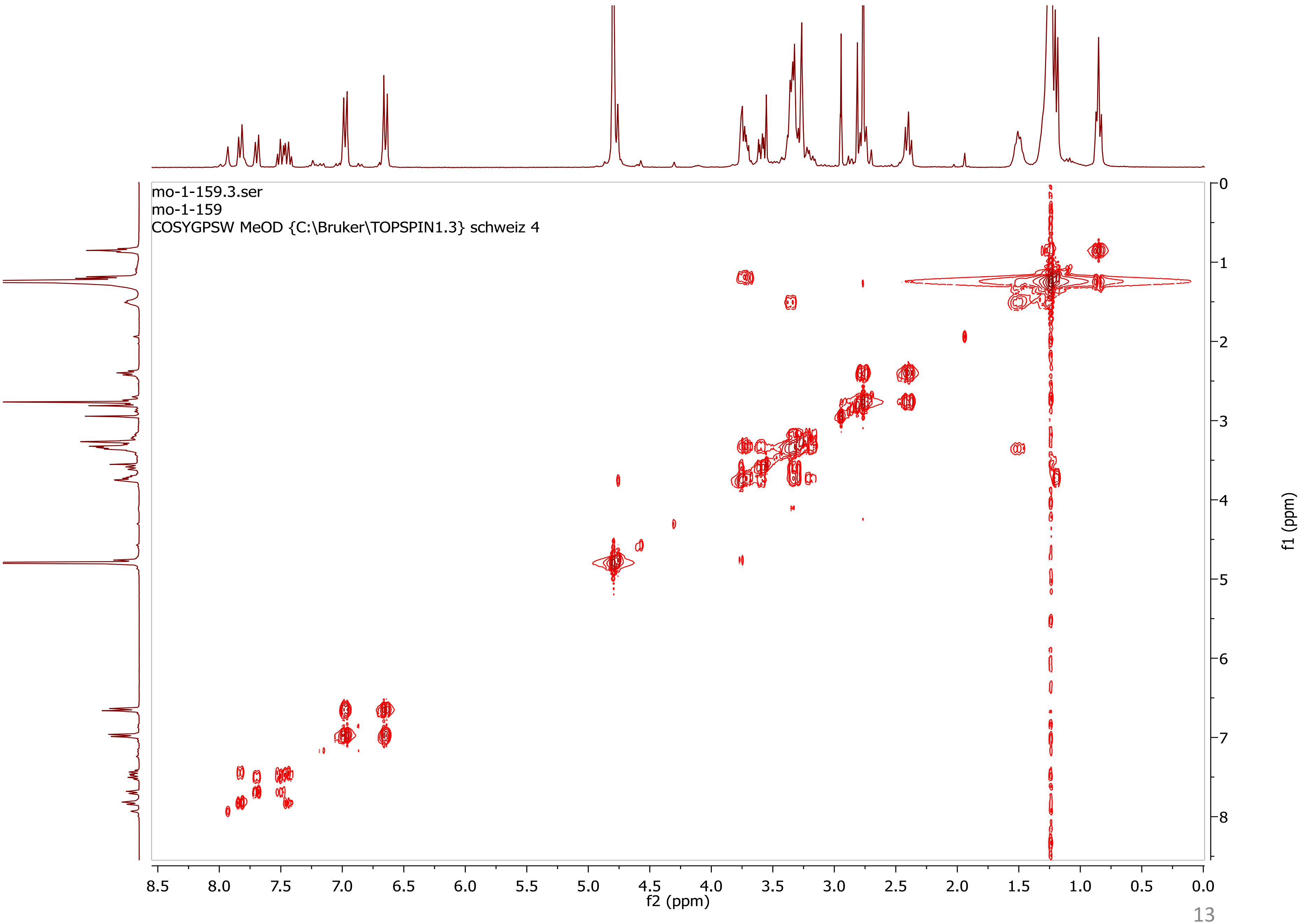
mo-1-159.3.ser
mo-1-159
COSYGPSW MeOD {C:\Bruker\TOPSPIN1.3} schweiz 4
f2 (ppm)
f1 (ppm)
8.5
8.0
7.5
7.0
6.5
6.0
5.5
5.0
4.5
4.0
3.5
3.0
2.5
2.0
1.5
1.0
0.5
0.0
0
1
2
3
4
5
6
7
8

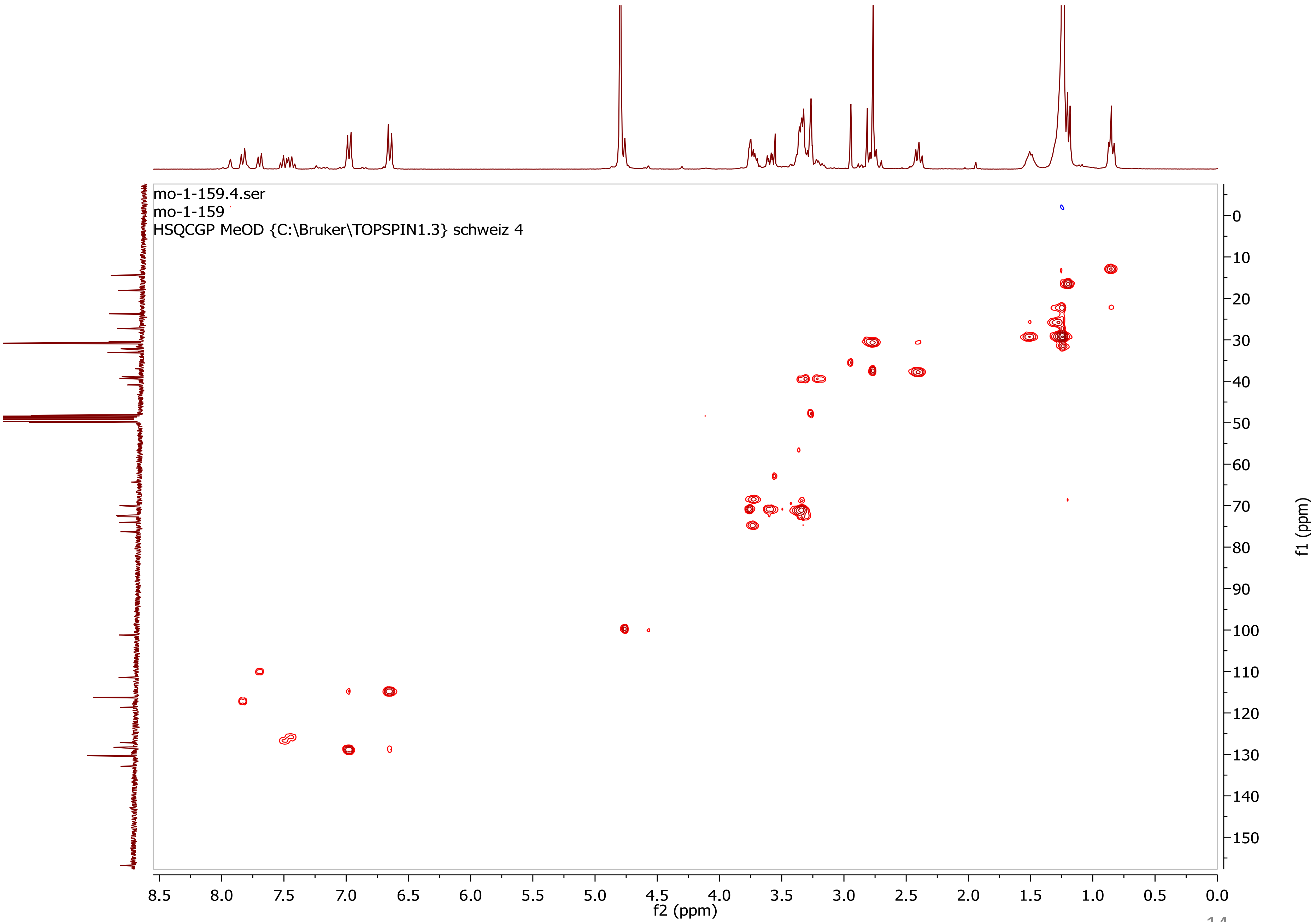
mo-1-159.4.ser
mo-1-159
HSQCGP MeOD {C:\Bruker\TOPSPIN1.3} schweiz 4
f2 (ppm)
f1 (ppm)
8.5
8.0
7.5
7.0
6.5
6.0
5.5
5.0
4.5
4.0
3.5
3.0
2.5
2.0
1.5
1.0
0.5
0.0
0
10
20
30
40
50
60
70
80
90
100
110
120
130
140
150

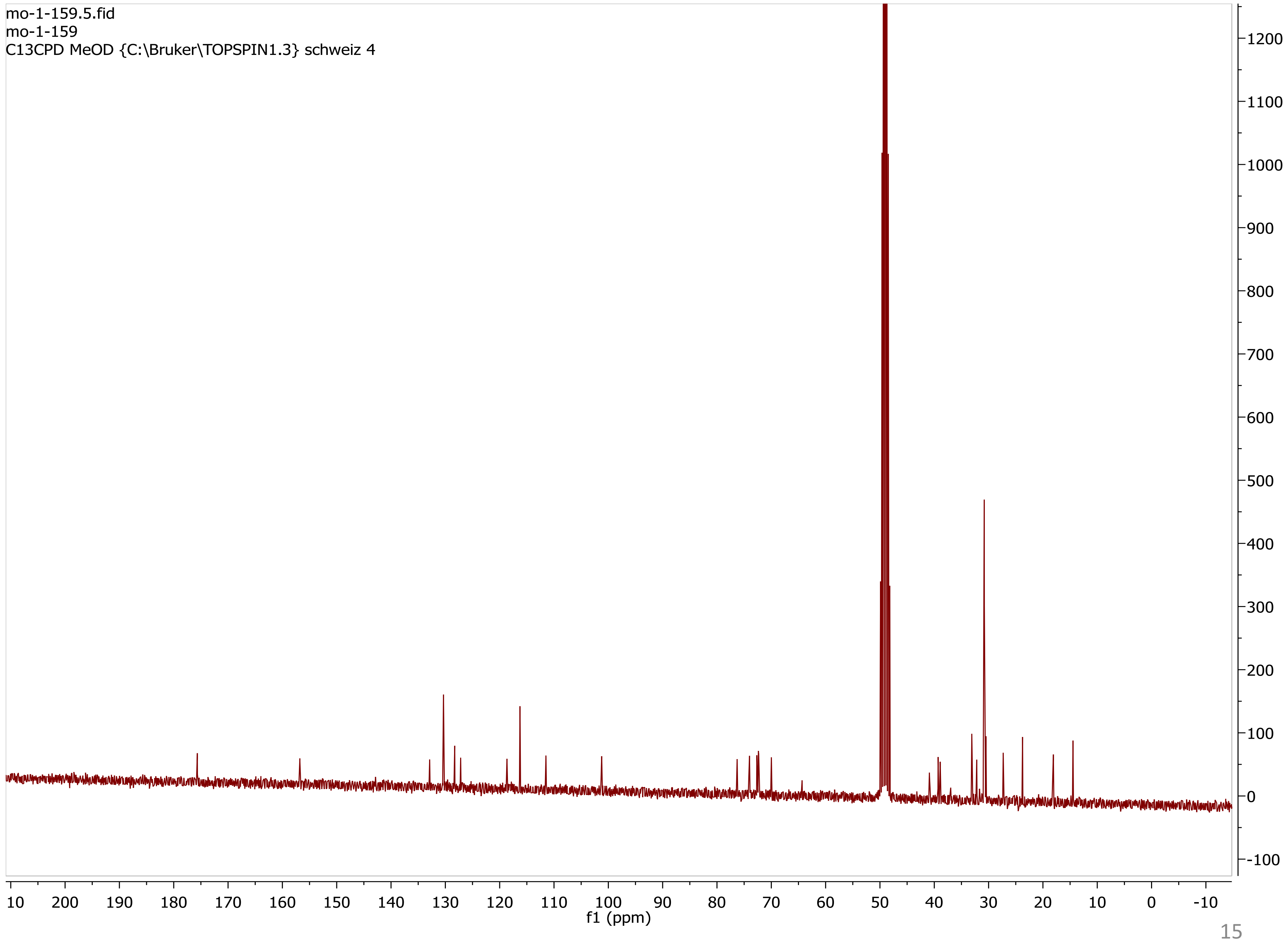
mo-1-159.5.fid
mo-1-159
C13CPD MeOD {C:\Bruker\TOPSPIN1.3} schweiz 4
f1 (ppm)

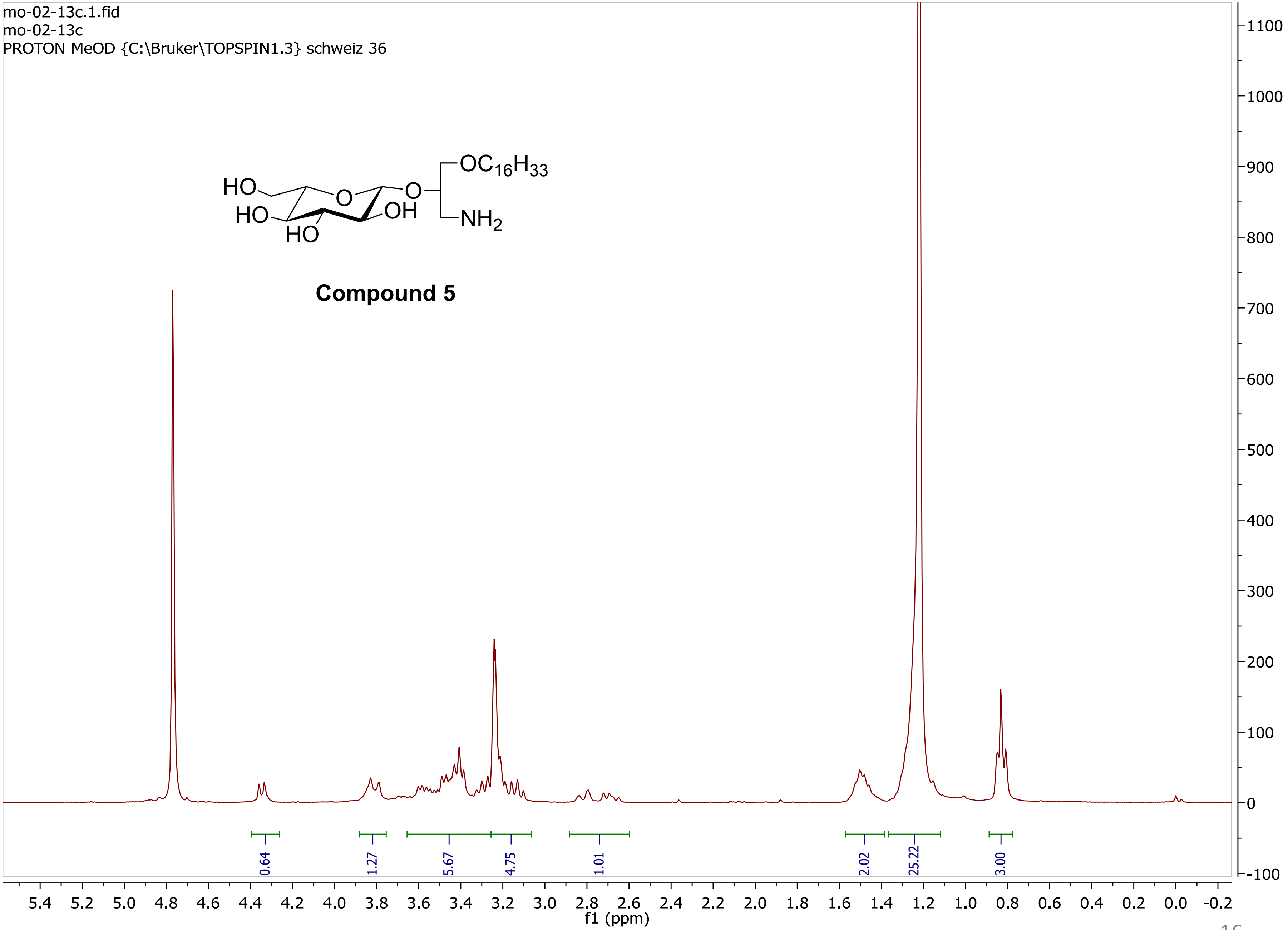
mo-02-13c.1.fid
mo-02-13c
PROTON MeOD {C:\Bruker\TOPSPIN1.3} schweiz 36
HO
HO
HO
O
OH
O
OC16H33
NH2
Compound 5
0.64
1.27
5.67
4.75
1.01
2.02
25.22
3.00
5.4 5.2 5.0 4.8 4.6 4.4 4.2 4.0 3.8 3.6 3.4 3.2 3.0 2.8 2.6 2.4 2.2 2.0 1.8 1.6 1.4 1.2 1.0 0.8 0.6 0.4 0.2 0.0 -0.2
f1 (ppm)
1100
1000
900
800
700
600
500
400
300
200
100
0
-100

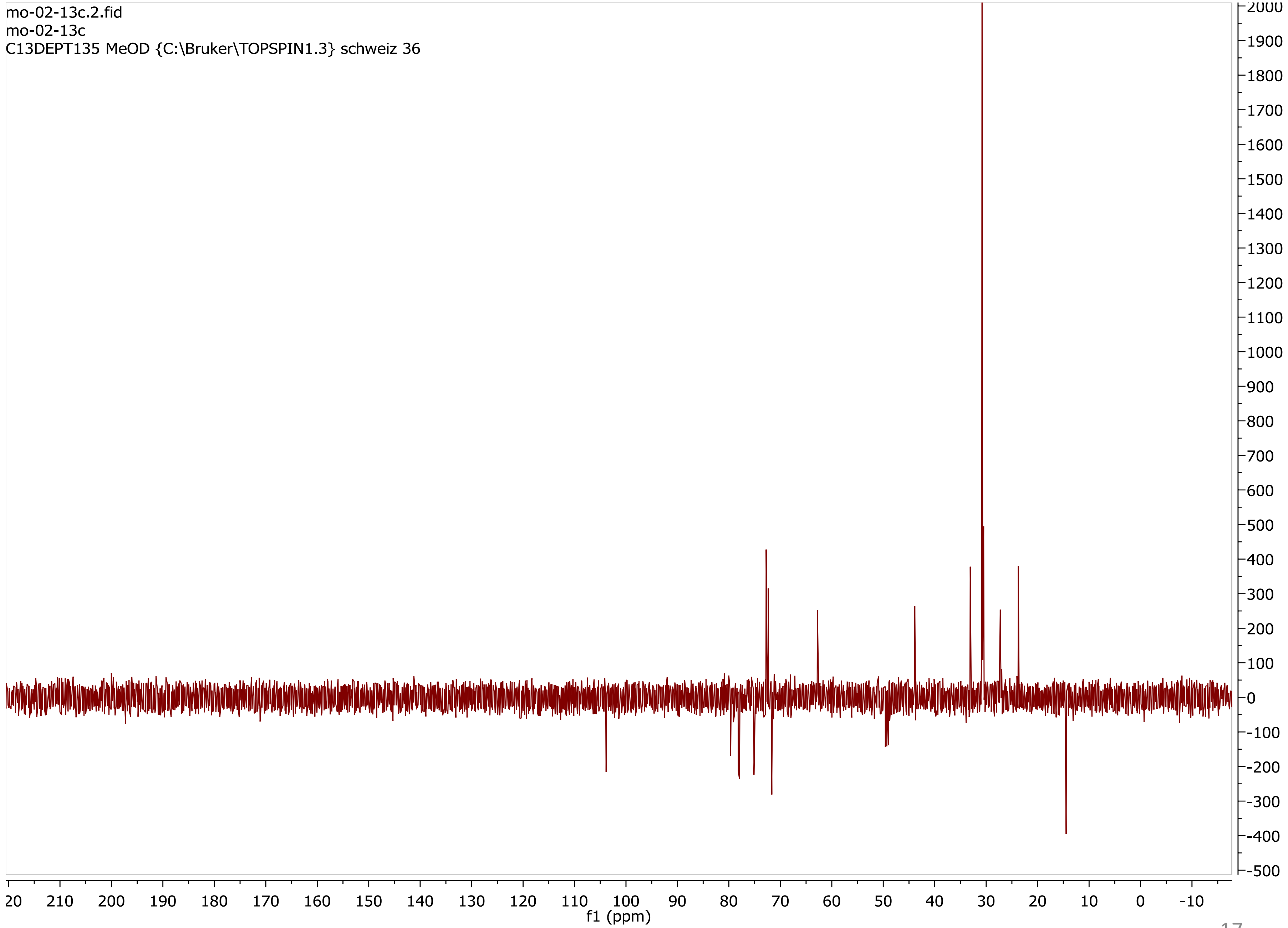
mo-02-13c.2.fid
mo-02-13c
C13DEPT135 MeOD {C:\Bruker\TOPSPIN1.3} schweiz 36
2000
1900
1800
1700
1600
1500
1400
1300
1200
1100
1000
900
800
700
600
500
400
300
200
100
0
-100
-200
-300
-400
-500
20
210
200
190
180
170
160
150
140
130
120
110
100
90
80
70
60
50
40
30
20
10
0
-10
f1 (ppm)

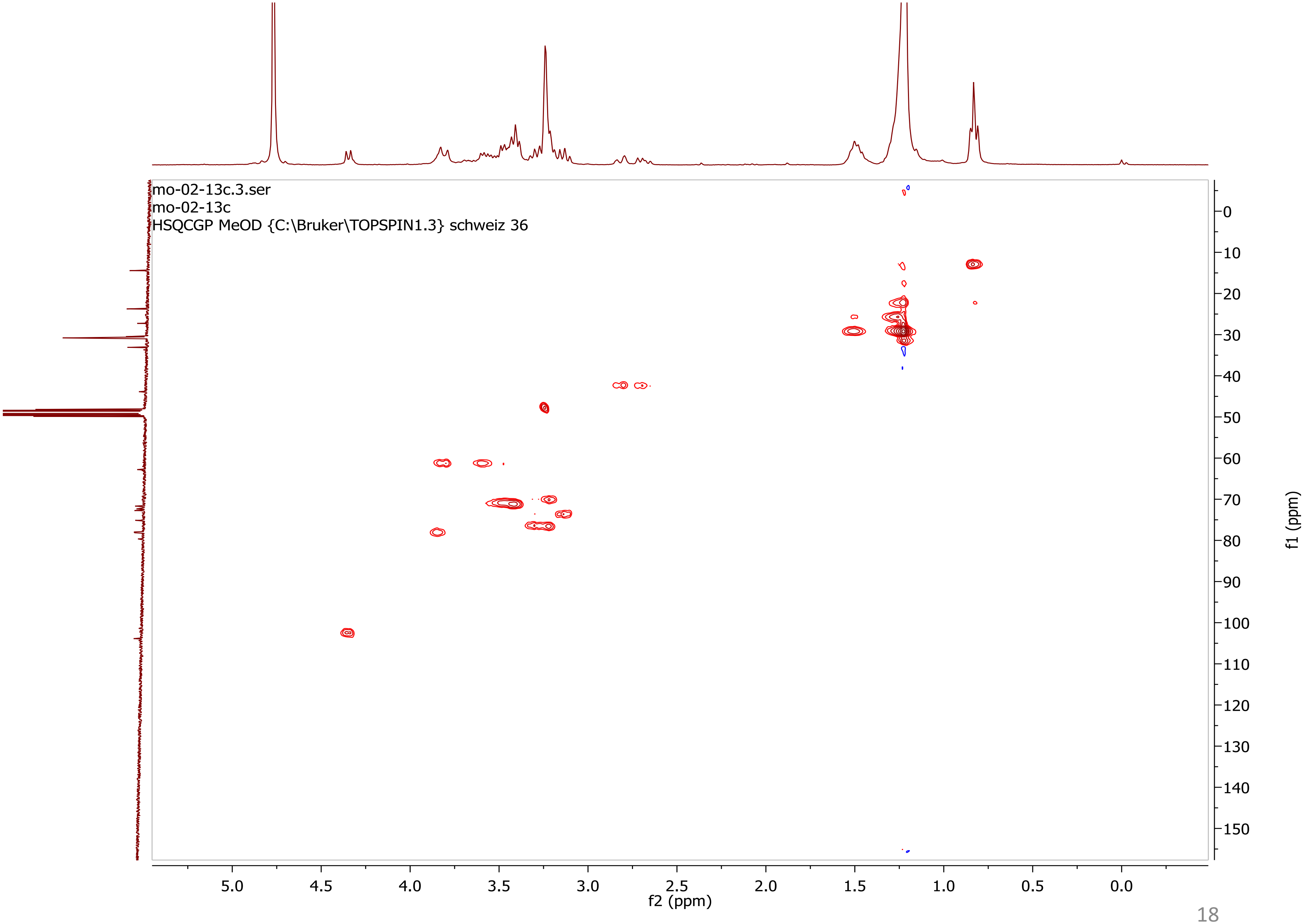
mo-02-13c.3.ser
mo-02-13c
HSQCGP MeOD {C:\Bruker\TOPSPIN1.3} schweiz 36
f2 (ppm)
f1 (ppm)
5.0
4.5
4.0
3.5
3.0
2.5
2.0
1.5
1.0
0.5
0.0
0
10
20
30
40
50
60
70
80
90
100
110
120
130
140
150

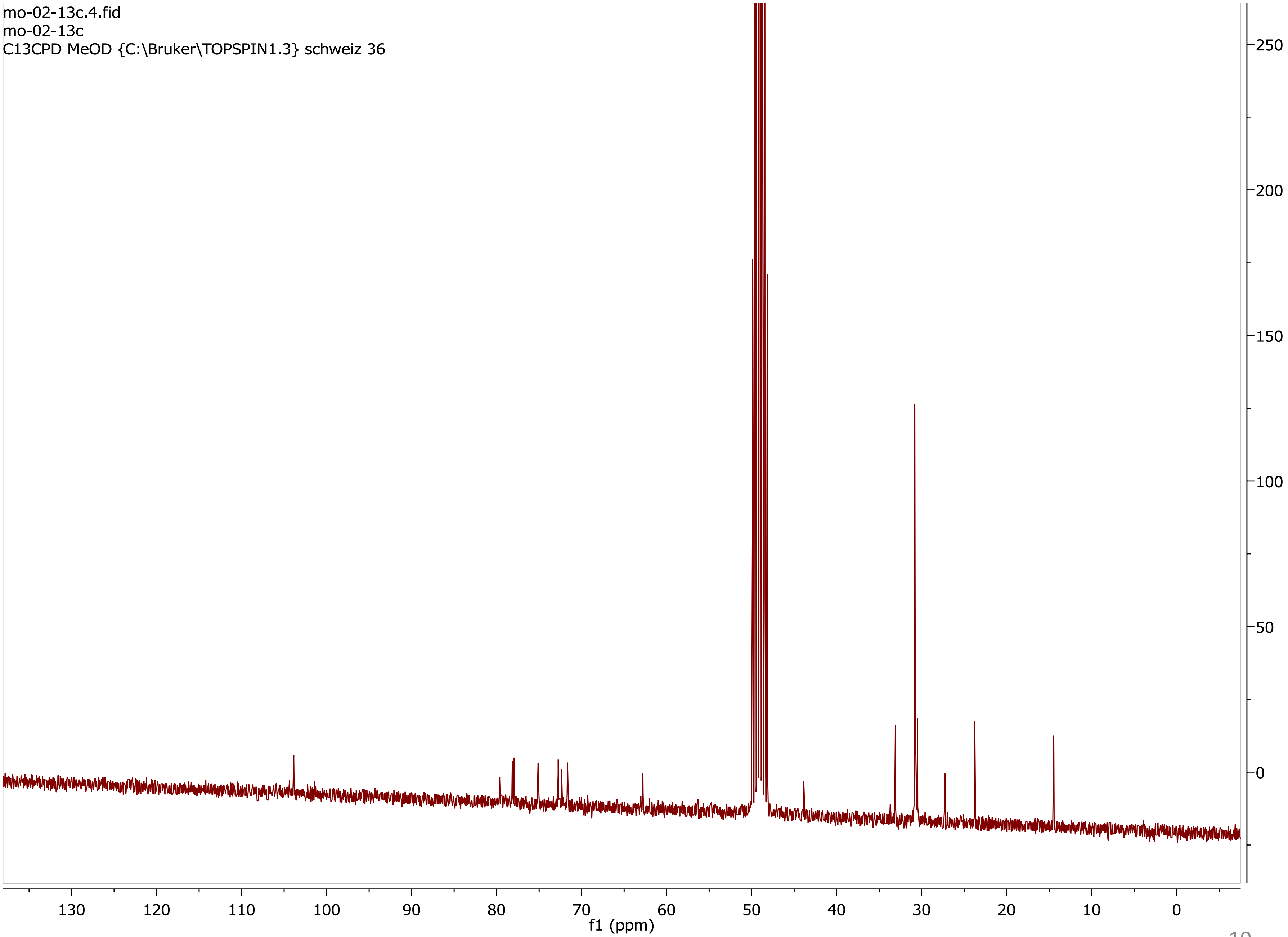
mo-02-13c.4.fid
mo-02-13c
C13CPD MeOD {C:\Bruker\TOPSPIN1.3} schweiz 36
250
200
150
100
50
0
130
120
110
100
90
80
70
60
50
40
30
20
10
0
f1 (ppm)

mo-02-07C.1.fid
mo-02-07C
PROTON MeOD {C:\Bruker\TOPSPIN1.3} schweiz 56

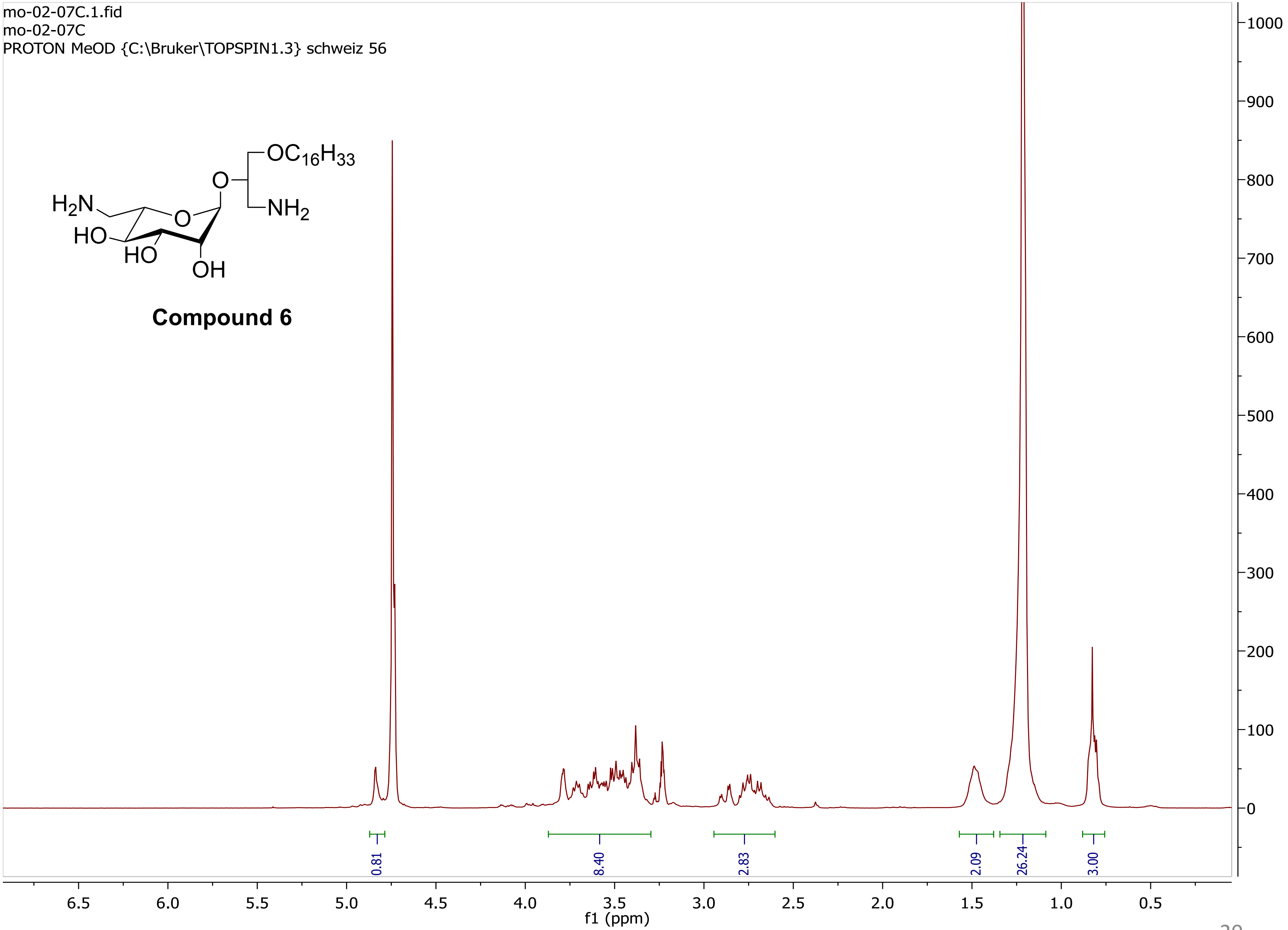

Compound 6

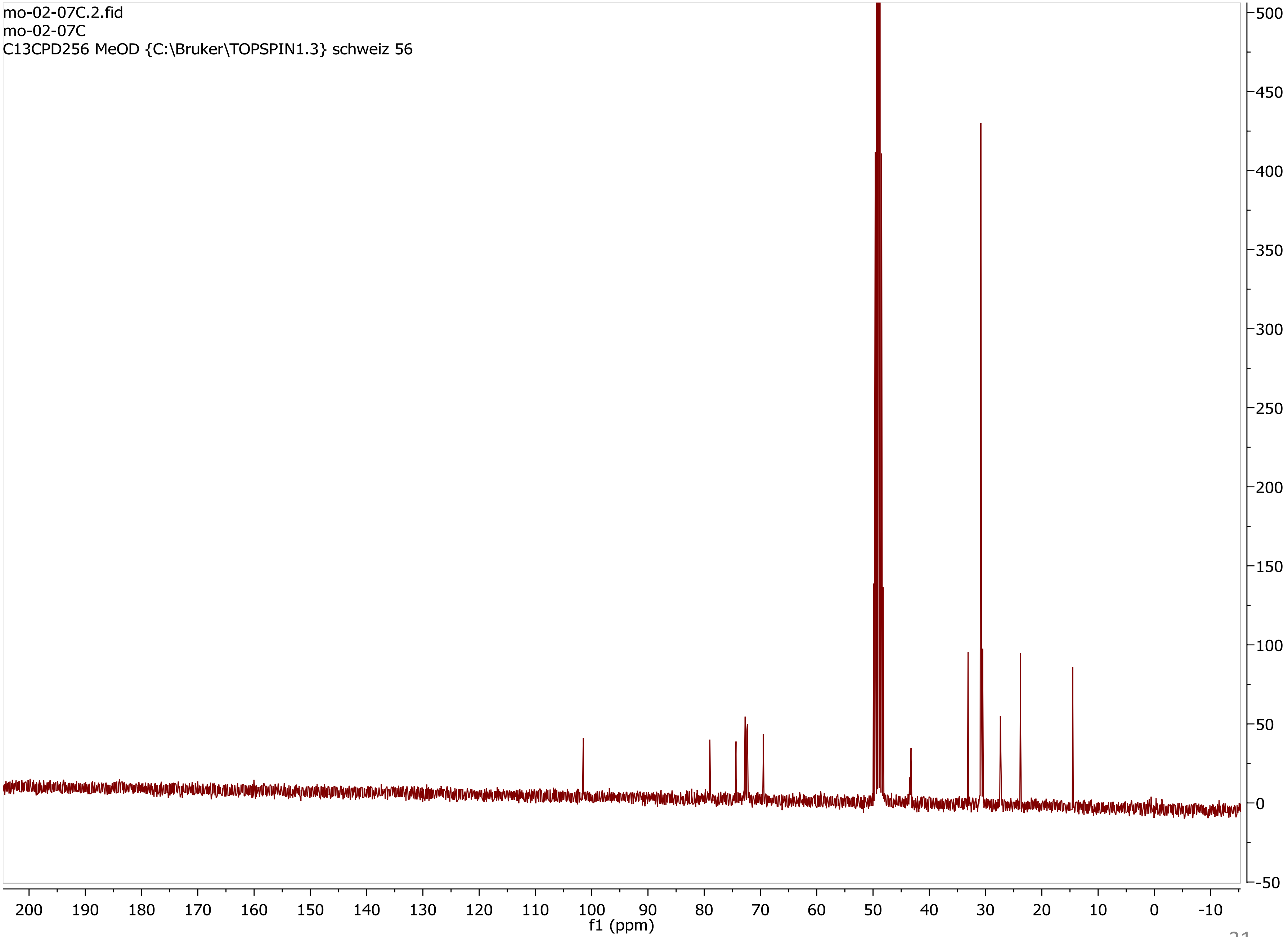
mo-02-07C.2.fid
mo-02-07C
C13CPD256 MeOD {C:\Bruker\TOPSPIN1.3} schweiz 56
500
450
400
350
300
250
200
150
100
50
0
-50
200
190
180
170
160
150
140
130
120
110
100
90
80
70
60
50
40
30
20
10
0
-10
f1 (ppm)

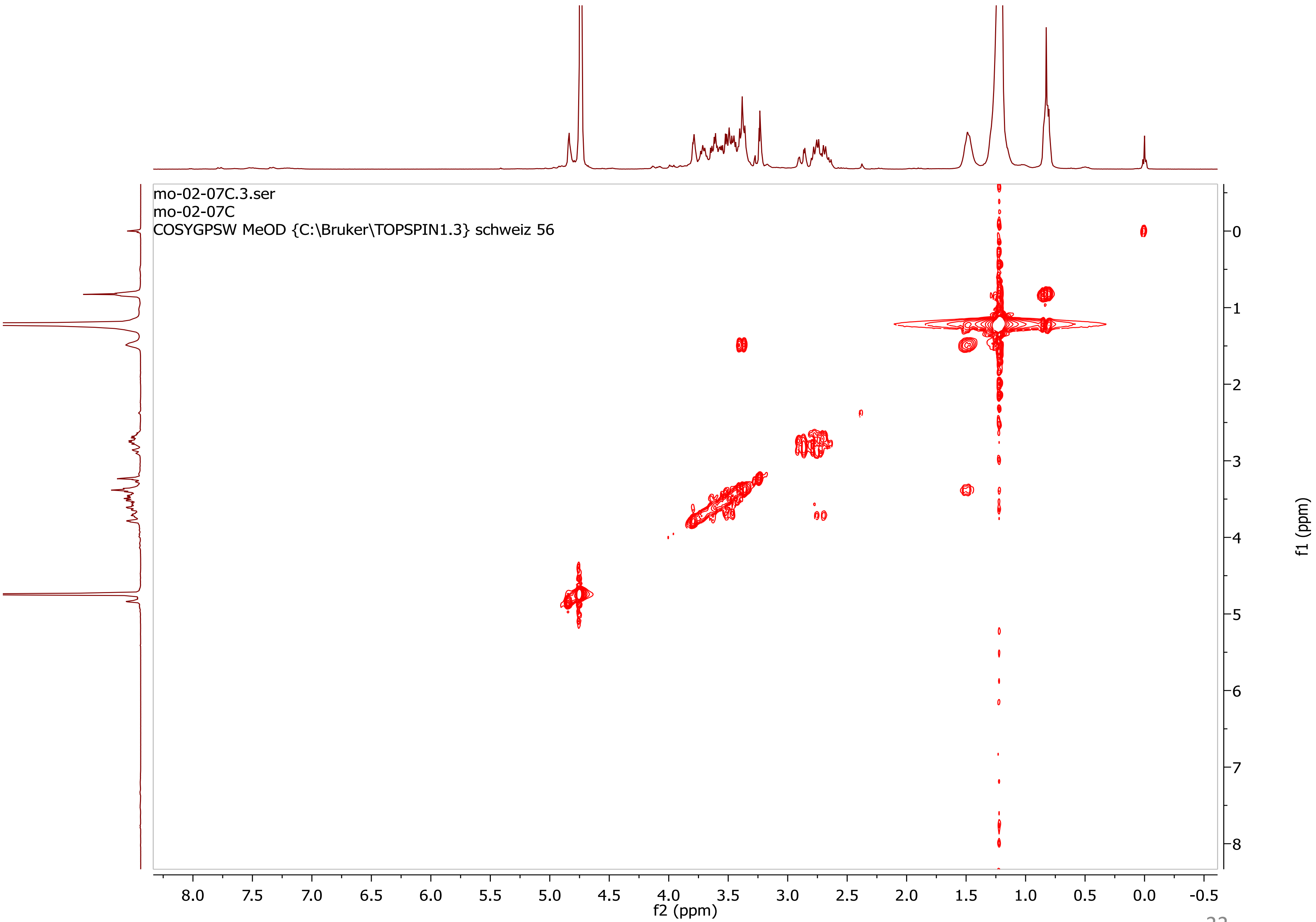
mo-02-07C.3.ser
mo-02-07C
COSYGPSW MeOD {C:\Bruker\TOPSPIN1.3} schweiz 56
8.0
7.5
7.0
6.5
6.0
5.5
5.0
4.5
4.0
3.5
3.0
2.5
2.0
1.5
1.0
0.5
0.0
-0.5
f2 (ppm)
0
1
2
3
4
5
6
7
8
f1 (ppm)

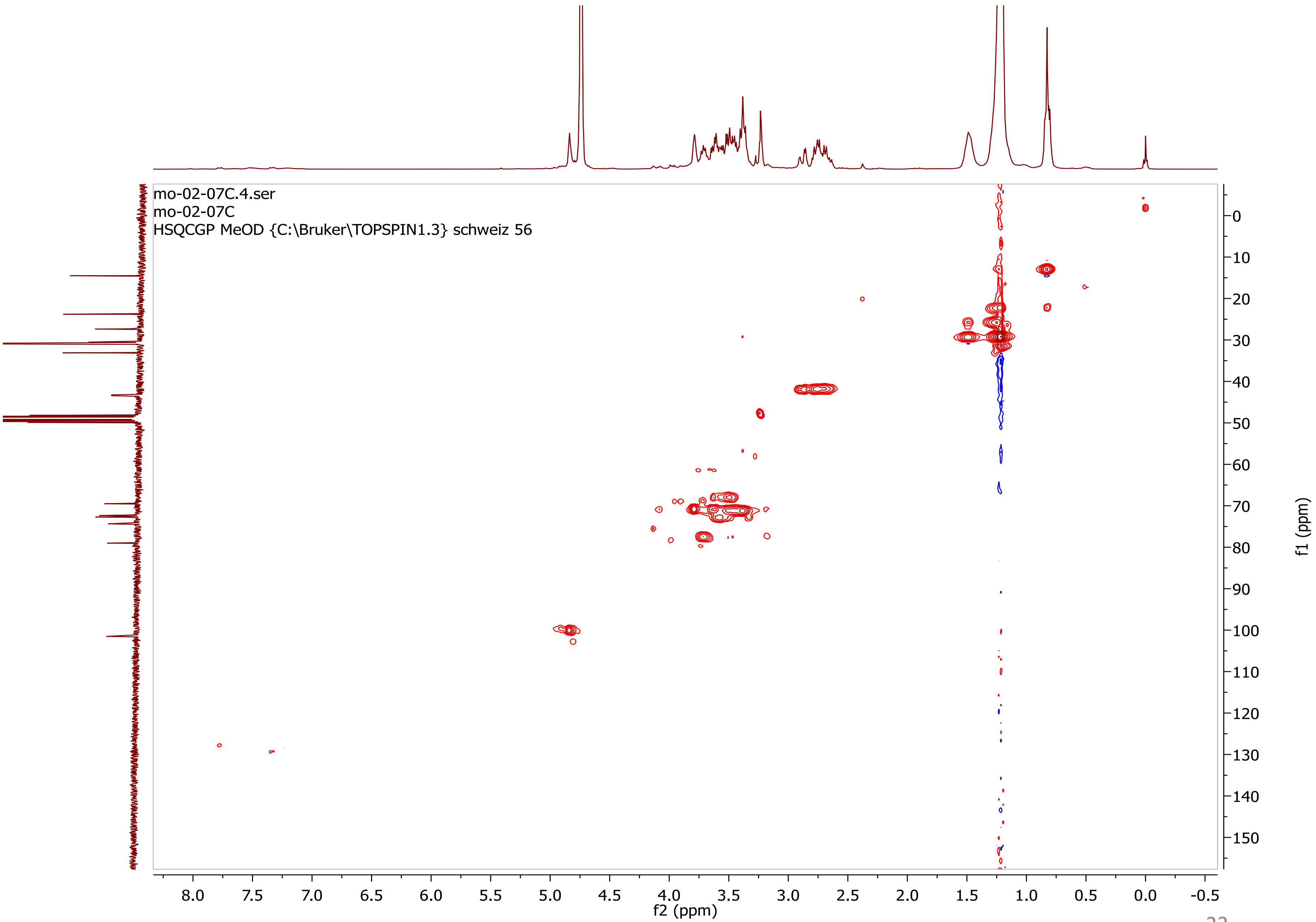
mo-02-07C.4.ser
mo-02-07C
HSQCGP MeOD {C:\Bruker\TOPSPIN1.3} schweiz 56
f2 (ppm)
f1 (ppm)
8.0
7.5
7.0
6.5
6.0
5.5
5.0
4.5
4.0
3.5
3.0
2.5
2.0
1.5
1.0
0.5
0.0
-0.5
0
10
20
30
40
50
60
70
80
90
100
110
120
130
140
150

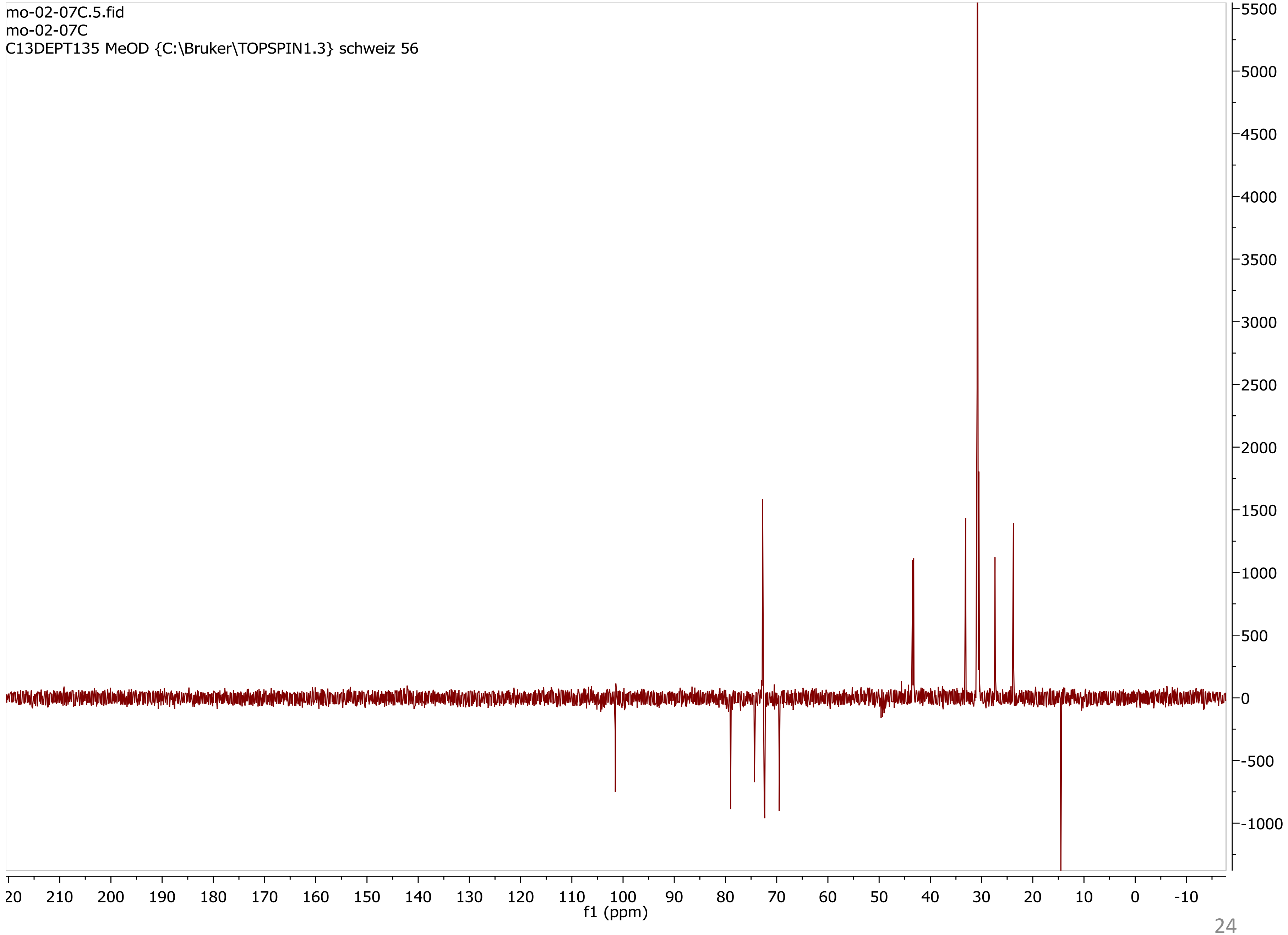
mo-02-07C.5.fid
mo-02-07C
C13DEPT135 MeOD {C:\Bruker\TOPSPIN1.3} schweiz 56
f1 (ppm)
